# Extracellular expression of a novel β-agarase from *Microbulbifer* sp. Q7, isolated from the gut of sea cucumber

**DOI:** 10.1186/s13568-017-0525-8

**Published:** 2017-12-19

**Authors:** Qian Su, Tianyi Jin, Yuan Yu, Min Yang, Haijin Mou, Li Li

**Affiliations:** College of Food Science and Engineering, Ocean University of China, Qingdao, 266003 Shandong China

**Keywords:** β-Agarase, *Microbulbifer* sp. Q7, Extracellular expression, Neoagaro-oligosaccharides

## Abstract

A novel endo-type β-agarase was cloned from an agar-degrading bacterium, *Microbulbifer* sp. Q7 (CGMCC No. 14061), that was isolated from sea cucumber gut. The agarase-encoding gene, *ID2563*, consisted of 1800 bp that encoded a 599-residue protein with a signal peptide of 19 amino acids. Sequence analysis suggested that the agarase belongs to the GH16 family. The agarase was expressed in *Escherichia coli* with a total activity of 4.99 U/mL in fermentation medium. The extracellular enzyme activity accounted for 65.73% of the total activity, which indicated that the agarase can be extracellularly secreted using the wild-type signal peptide from *Microbulbifer* sp. Q7. The agarase exhibited maximal activity at approximately 40 °C and pH 6.0. It was stable between pH 6.0 and pH 9.0, which was a much wider range than most of the reported agarases. The agarase was sensitive to some metal ions (Cu^2+^, Zn^2+^ and Fe^3+^), but was resistant to urea and SDS. The agarase hydrolyzed β-1,4-glycosidic linkages of agarose, primarily yielding neoagarotetraose and neoagarohexaose as the final products. These indicate that this recombinant agarase can be an effective tool for the preparing functional neoagaro-oligosaccharides.

## Introduction

Agarose consists of alternating 3-*O*-linked β-d-galactose and 4-*O*-linked 3,6-anhydro-α-l-galactose units (Duckworth and Yaphe 1971). Oligosaccharides prepared from agarose exhibit various biological and physiological functions, such as antioxidant (Wang et al. 2004), and anti-inflammatory activities (Yun et al. 2013), moisturizing and whitening effects on melanoma cells (Kobayashi et al. 1997), inhibition of bacterial growth, and inhibition of starch degradation (Giordano et al. 2006). Owing to these properties of agar-oligosaccharides, their preparation and potential applications have attracted widespread attention.

Enzymatic hydrolysis is one of the most widely used methods for the preparation of agar-oligosaccharides owing to its high specificity and efficiency. Agarases are a group of glycoside hydrolases (GH), which includes α-agarases (EC 3.2.1.158) and β-agarases (EC 3.2.1.81). These enzymes cleave α-1, 3-linkages of agarose to produce agaro-oligosaccharides (Potin et al. 1993) and inter-β-1,4-linkages to produce neoagaro-oligosaccharides (Kirimura et al. 1999), respectively. Some agarases, mostly belonging to β-agarase, have been isolated and cloned from different microorganisms, such as *Vibrio* sp. AP-2 (Aoki et al. 1990), *Alteromonas* sp. E-1 (Kirimura et al. 1999), *Bacillus* sp. MK03 (Suzuki et al. 2003), *Zobelli*a *galactanivorans* Dsij (Allouch et al. 2003), the archaea *Halococcus* sp. 197A (Minegishi et al. 2013), *Thalassospira profundimonas* sp. fst-13007 (Zeng et al. 2016), *Gayadomonas joobiniege* sp. G7 (Jung et al. 2017) and *Aquimarina agarilytica* sp. ZC1 (Lin et al. 2017).

Sea cucumbers are deep-sea echinoderms that feed on seaweed and plankton. Thus, we hypothesized that their guts contain bacterial strains that degrade seaweed polysaccharides. In our previous work, we isolated a strain, *Microbulbifer* sp. Q7, from sea cucumber gut that was able to degrade agar and alginate. The whole genome of *Microbulbifer* sp. Q7 was sequenced and the agarase gene was identified (Yang et al. 2017). Herein, we describe the heterologous expression of agarase in *E. coli.* To increase extracellular secretion of the agarase, the wild-type signal peptide from *Microbulbifer* sp. Q7 was used. Its enzymatic activity and hydrolytic products of the agarase were also studied.

## Materials and methods

### Strains, plasmids and media

*Microbulbifer* sp. Q7 (CGMCC no. 14061) was isolated from the guts of sea cucumbers and cultured in 2216E medium, the whole genome of Q7 was sequenced using a HiSeq PE150 (Novogene Bioinformatics Technology Co. Ltd., China) (Yang et al. 2017). The *E. coli* DH5α were used for cloning, and the *E. coli* BL21(DE3) were used for protein expression. Both strain were cultured in Luria–Bertani (LB) medium containing 100 μg/mL ampicillin. The pProEX-HTa vector (Yellow Sea Fisheries Research Institute, Chinese Academy of Fishery Science) was used as the cloning and expression vector.

### Sequence analysis of the *ID2563* gene

Our previous work sequenced the *Microbulbifer sp.* Q7 genome and identified the agarase gene, *ID2563* (Yang et al. 2017). The *ID2563* sequence was deposited into NCBI under the Accession Number WP_066965750.1.DNAMAN software was used to analyze the sequence of the gene. The signalP 4.1 server (http://www.cbs.dtu.dk/services/SignalP/) was used to predict the signal peptide sequence of *ID2563*. Analysis of its physicochemical properties was performed using ProtParam (http://web.expasy.org/protparam/). The conserved domain and catalytic site were predicted by the Conserved Domain Database (https://www.ncbi.nlm.nih.gov/cdd/). Mega5.0 was used to construct a phylogenetic tree using the neighbor-joining method.

### Cloning and expression of the recombinant agarase

Genomic DNA from *Microbulbifer* sp. Q7, was extracted using a total DNA extraction kit (Sangon, Shanghai China). For extracellular agarase expression, the *ID2563* gene with its original signal sequence was amplified by using the following primers, *ID2563*-F (CGGGATCCATGAAAACCACTCAGGGCG, *Bam*HI site underlined) and *ID2563*-R (CCCAAGCTTTTAATTACTTAGCACGAACTTATCC, *Hin*dIII site underlined). The amplicon was cloned into pProEX-HTa. The recombinant plasmid was transformed into *E. coli* DH5α and plated on LB supplemented with 100 μg/mL ampicillin. Successful cloning of *ID2563* into pProEX-HTa was confirmed by sequencing. pProEX-HTa-*ID2563* was transformed into *E. coli* BL21 (DE3) and plated on LB supplemented with 100 μg/mL ampicillin. For agarase expression, the *E. coli* BL21(DE3) containing pProEX-HTa-*ID2563* were grown at 37 °C in LB medium supplemented with 100 μg/mL ampicillin. When the OD_600_ reached 0.6–0.8, isopropyl-β-thiogalactoside (IPTG) was added to a final concentration of 1 mM. Cell were incubated at 23 °C 160 rpm for 24 h.

### Assay of enzyme activity

Agarase activity was determined using the 3,5-dinitrosalicylic acid (DNS) method (Miller 1959). Briefly, 100 μL of enzyme was added to 900 μL of 20 mM Tris–HCl pH 7.2 containing 0.2% (w/v) agarose, and the reaction was incubated at 40 °C for 5 min. One milliliter of DNS reagent added, and the reaction was heated in boiling water for 5 min and rapidly cooled. The absorbance was measured at 520 nm and compared with a standard curve for d-galactose. Enzyme activity (1 U) was defined as the amount of enzyme required to liberate 1 μM of d-galactose per min.

### Purification of recombinant agarase

BL21(DE3) *E. coli* expressing his-tagged agarase were pelleted by centrifugation (10,000 rpm, 10 min). The supernatant and pellet were used to determine extracellular and intracellular agarase activity, respectively. Pelleted cells were resuspended in phosphate buffer saline and lysed by ultrasonication. Cell debris was removed by centrifugation (12,000 rpm, 10 min). Extracellular his-tagged agarase was purified using a Ni Sepharose 6FF column (GE Healthcare, USA) and imidazole concentrations between 10 and 400 mM. Fractions that were positive for agarase activity were pooled and concentrated using an ultrafiltration concentrator. Purified agarase was detected by 12% sodium dodecyl sulfate polyacrylamide gel electrophoresis (SDS-PAGE). Agarase concentration was measured using the prestained protein ladder (Thermo, range 10–180 kDa).

### Native-PAGE and zymogram analysis

Native-PAGE of the purified recombinant agarase solution was performed on 10% gel at 4 °C. Zymogram analysis gel was soaked in Tris–HCl buffer (50 mM, pH 7.0) for 5 min after the native-PAGE. Then the gel was overlaid onto a sheet of 2% (w/v) agarose in Tris–HCl buffer (50 mM, pH 7.0) and incubated at 40 °C for 30 min. To visualize agarase activity, the agarose sheet was flooded with Lugol’s iodine solution. Then the gel was removed from the agarose sheet and stained with Coomassie Brilliant Blue R-250.

### Properties of enzyme

Agarase activity was measured at six temperatures between 30 and 60 °C under the standard conditions to determine the optimum temperature for activity. The thermal stability of agarase was determined based on its enzymatic activity after pre-incubation at different temperatures.

The optimum pH for agarase was determined by assessing its activity at different pH values. Three buffers was used: 50 mM Na_2_HPO_4_-citric acid (pH 3.0, 4.0 and 5.0), 50 mM sodium phosphate (pH 6.0, 7.0 and 8.0), 50 mM Tris–HCl (pH 9.0) and 50 mM Na_2_CO_3_-NaOH buffer (pH 10.0 and 11.0). Extracellular agarase was pre-incubation in the buffers listed above for 2 h at 20 °C and activity was measured to determine pH-dependent stability.

To determine the effects of ions and other molecules on agarase activity, the assay was performed in the presence of the following reagents: Na^+^ and Fe^2+^ (5, 20 and 50 mM), K^+^, Mg^2+^, Ca^2+^, Li^+^, Fe^3+^, Zn^2+^, Cu^2+^, EDTA, SDS, DTT and Urea (5 mM), 0.5% (v/v) ethylene thioglycol, 0.5% (v/v) Tween-80 and 0.5% (v/v) Triton-100. Enzyme activity was measured at 42 °C and pH 7.0. Reactions in the absence of the additives were used as controls.

### Analysis of enzymatic hydrolytic products

The appropriate concentration of agarase was incubated with 1% agarose at 37 °C for approximately 6 h. Then solution was separated from the undegraded agarose by centrifugation. Macromolecular agarose and impurities were precipitated from the supernatant using different ratios of alcohol to supernatant (the maximum ratio is 6:1). The end products, the supernatant of the maximum ratio precipitation, were freeze-dried (FD-1A-50 vacuum freezer dryer, Xi An DP Biological Technology, China) for use in further experiments.

The molecular masses of the end products were detected by using electrospray ionization mass spectrometry (ESI–MS). Agar-oligosaccharide samples were dissolved in acetonitrile/1 mM NH_4_HCO_3_ (1:1, v/v) and analyzed with the micromass Q-TOF and Q-TOF ultima instruments (Waters, Manchester, UK) in negative-ion mode.

The end products were also analyzed by ^13^C-NMR (carbon-13 nuclear magnetic resonance) spectroscopy. The lyophilized powder was dissolved in D_2_O and spectra were recorded on an Agilent ProPulse 500 MHz NMR system. MestRe Nova software was used to analyze the ^13^C-NMR results. Deuterated acetone was used as an internal standard.

Agarase was added to 30 mL of 20 mM Tris–HCl pH 7.2 containing 1% (w/v) agarose. The reaction were incubated at 40 °C for 0–5 h and quenched by boiling for 5 min to denature the agarase. Subsequently, the reaction products were applied to a thin layer chromatography (TLC) silica gel 60 F254 plate (Merck, Darmstadt, Germany) using a solution of *n*-butyl alcohol/acetic acid/distilled water (2:1:1, v/v/v) as the mobile phase. The plate was sprayed with 10% H_2_SO_4_ in alcohol and heated to 110 °C for 10 min to visualize the product spots. To determine the composition of the final oligosaccharide products, the first two spots on the TLC plate were removed. Spots were dissolved in a small volume of distilled water and analyzed by ESI–MS (Agilent Technologies 6460 Triple Quad LC/MS) to determine their molecular weights.

## Results

### Sequence analyses of the *ID2563* gene

**Fig. 1:**
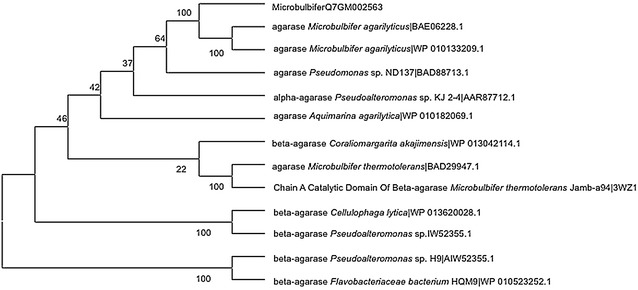
The phylogenetic tree of the recombinant agarase

A single open-reading frame composed of 1800 bp was obtained directly from the genomic DNA of *Microbulbifer* sp. Q7. The anticipated protein product of the *ID2563* gene comprised 599 amino acids and contained an N-terminal signal peptide that was 19 residues in length (M_1_-K_2_-T_3_-T_4_-Q_5_-G_6_-A_7_-L_8_-A_9_-A_10_-L_11_-V_12_-F_13_-S_14_-T_15_-P_16_-L_17_-M_18_-A_19_). The SignalP 4.1 server suggested the cleavage site of the signal peptide was likely between Ala_19_ and Ala_20_. Based on the agarase sequence, a molecular mass (Mw) of 64.6 kDa and an isoelectric point of 4.47 were calculated using ProtParam. The NCBI’s Conserved Domain Database, classified the agarase protein as belonging to the GH16 family. Eleven active sites residues (N_69_-W_71_-W_137_-S_143_-E_146_-D_148_-E_151_-F_174_-R_176_-E_256_-Q_258_) and three catalytic sites residues (E_146_-D_148_-E_151_) were found within 545 aa. Protein BLAST of the amino acid sequence showed that the protein shared 85% homology with the agarase from *Microbulbifer agarilyticus* (GenBank Accession No. BAE06228.1). There were few similarities with the agarase sequences of *Coraliomargarita akajimensis* (WP _013042114.1, 56%), *Cellvbrio* sp. BP (EIK45872.1, 48%) and *Reichenbachiella agariperforans* (SHK69922.1, 44%). The phylogenetic tree indicating the relationship between the *ID2563* protein product with agarases from other microorganisms is shown in Fig. 1.

### Expression and purification of the agarase

**Fig. 2:**
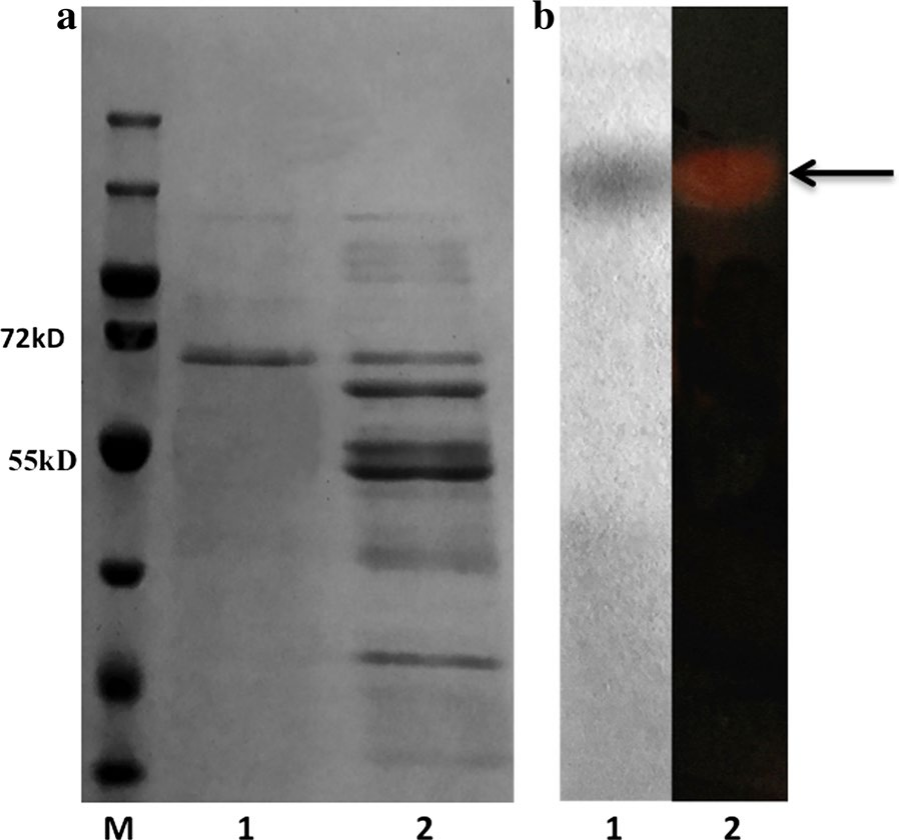
SDS-PAGE and zymogram analysis of purified recombinant agarase. **a** SDS-PAGE analysis of the purified recombinant agarase. Lane M: protein markers. Lane 1: purified agarase. Lane 2: extracellular protein components. **b** Native-PAGE analysis of the purified agarase. Lane 1: purified agarase protein stained with Coomassie brilliant blue R-250. Lane 2: zymogram of the purified agarase. The native-PAGE gel was overlaid onto a sheet containing 2% (w/v) agarose in Tris–HCl buffer (50 mM, pH 7.0), incubated for 30 min at 40 °C, and then incubated with Lugol’s iodine solution to visualize agarase activity

The sequence of the *ID2563* gene with the wild-type signal peptide was amplified using the primers *ID2563*-F/*ID2563*-R and cloned into a modified pProEX-HTa vector to produce recombinant agarase with an N-terminal histiding tag. The recombinant protein was soluble and found in both the supernatant and cell extracts. Its activity in 1 mL of culture broth was 3.28 U from the supernatant fraction and 1.17 U in the cell extract fraction. The total activity, 4.99 U/mL of culture broth, was 8.6 times higher than that of *Microbulbifer* sp. Q7 (0.58 U/mL). Extracellular agarase was purified using a Ni Sepharose 6FF column and was approximately 65 kDa (Fig. 2a), consistent with the theoretical molecular mass. Zymogram analysis showed a single protein band with agarase activity.

### Enzymatic properties of the recombinant agarase

**Fig. 3:**
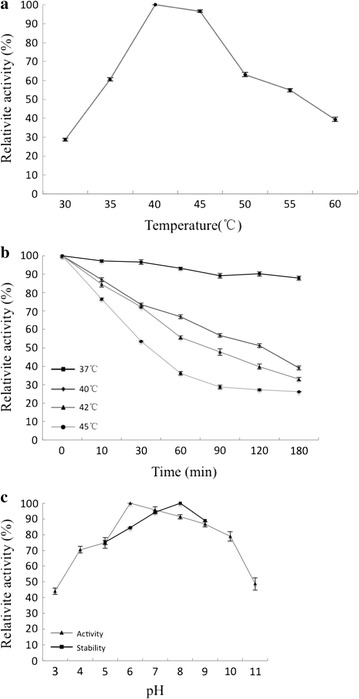
Characterization of the recombinant agarase. **a** The effect of temperature on agarase activity. **b** Thermal stability of recombinant agarase. The agarase was pre-incubated at various temperatures, and remaining activity was measured at 42 °C. **c** The optimal pH and pH stability of agarase was measured in 50 mM Na_2_HPO_4_-citric acid (pH 3.0, 4.0 and 5.0), 50 mM sodium phosphate (pH 6.0, 7.0 and 8.0), 50 mM Tris–HCl (pH 9.0) and 50 mM Na_2_CO_3_-NaOH (pH 10.0 and 11.0)

The effect of temperature on activity was examined by measuring the relative activity at various temperatures ranging from 30 to 60 °C. The highest activity was observed at 40 °C (Fig. 3a). Thermal stability experiments revealed agarase remained 90.12% of its activity at 37 °C for 2 h, greater than 55% activity at 40 and 42 °C for 1 h, and 36.16% activity at 45 °C for 1 h (Fig. 3b).

To study the effect of pH, agarase activity was measured in several buffers at 40 °C. Greater than 70% enzyme activity was observed for reactions between pH 4.0 and 10.0 with the maximum activity observed at pH 6.0. Agarase was stable over a broad pH range, particularly in mildly alkaline conditions (Fig. 3c).

Several mental ions and other molecules were added to the standard reaction system to determine their effect on agarase activity. Activity was normalized to the standard condition in the absence of additives. The agarase activity was strongly inhibited by Cu^2+^, Zn^2+^ and Fe^3+^, whereas 5 mM Na^+^ and Fe^2+^ slightly stimulated activity. Activity markedly increased in the presence of 0.5% β-mercaptoethanol (Table 1).

**Table 1:** Effects of ions and chemical reagents on agarase activity

Ions and chemical reagents	Concentrations	Relative activity (%)
Na^+^	5 mM	113.34 ± 0.57
	20 mM	97.93 ± 1.02
	50 mM	96.94 ± 0.75
	5 mM	116.46 ± 0.43
Fe^2+^	20 mM	82.75 ± 0.73
	50 mM	77.65 ± 0.82
Cu^2+^	5 mM	6.2 ± 0.45
K^+^	5 mM	97.26 ± 0.63
Ca^2+^	5 mM	96.78 ± 0.34
Zn^2+^	5 mM	14.97 ± 1.02
Mg^2+^	5 mM	76.92 ± 0.36
Li^+^	5 mM	92.00 ± 0.58
Fe^3+^	5 mM	34.65 ± 1.09
EDTA	5 mM	86.47 ± 1.44
SDS	5 mM	99.26 ± 0.78
DTT	5 mM	103.44 ± 0.99
Urea	5 mM	96.01 ± 0.73
Tween-80	0.5% (v/v)	89.72 ± 0.82
Triton-100	0.5% (v/v)	100.94 ± 0.69
β-Mercaptoethanol	0.5% (v/v)	141.05 ± 0.74

The data shown are representative of three independent experiments

The activity measured under the standard condition is defined as 100%

The concentration indicates the final reagents concentrations in the reaction

### Enzymatic product analysis

**Fig. 4:**
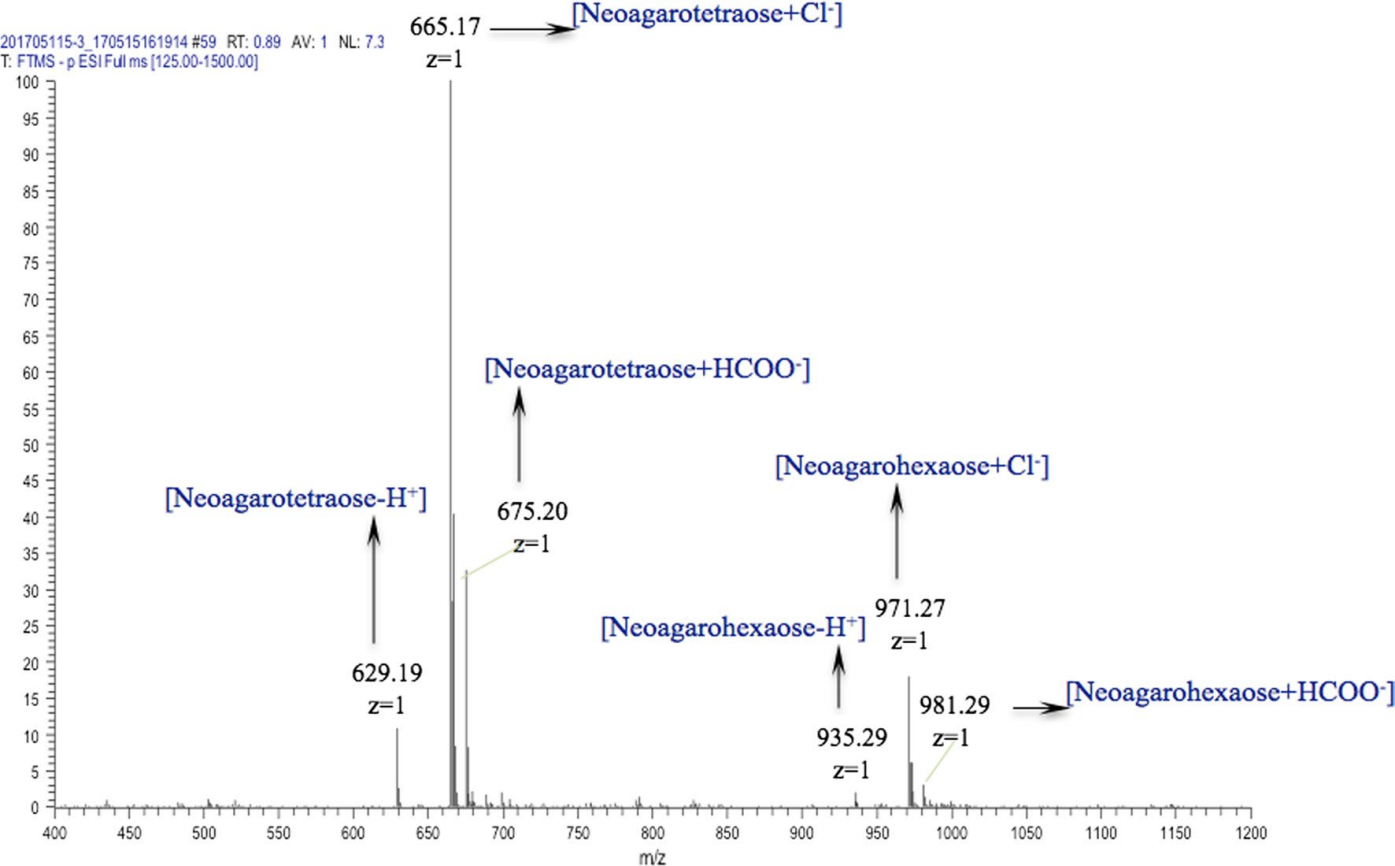
Determination of molecular masses of the hydrolytic products by mass spectrometry

**Fig. 5:**
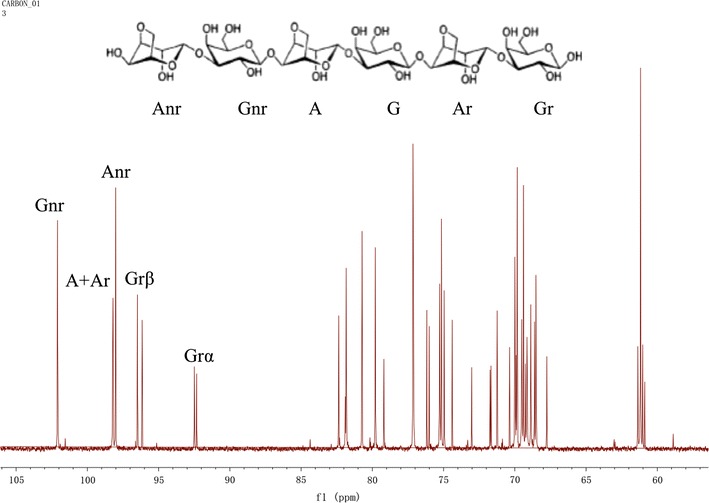
^13^C-NMR spectrum of the reaction products of the agarase. The upper formula is the structure of neoagarohexaose. Peak assignments are labeled according to the nomenclature defined in the upper formula. A and G refer to the 4-*O*-linked 3,6-anhydro-α-l-galactopyranose and 3-*O*-linked β-d-galactopyranose; r and nr denote the reducing and non-reducing end; α/β for anomer

Electrospray ionization mass spectrometry of the reaction product showed m/z peaks at 629.19 (M−H)^+^, 665.17 (M+Cl)^−^ and 675.20 (M+HCOO)^−^, corresponding to neoagarotetraose and m/z peaks at 935.29 (M−H)^−^, 971.27 (M+Cl)^−^ and 981.29 (M+HCOO)^−^, corresponding to neoagarohexaose (Fig. 4). The anomeric carbon of agar-oligosaccharides and neoagar-oligosaccharides have different chemical shift. Therefore, the presence of anomer carbon by ^13^C-NMR could identify which oligosaccharides products resulted from the reaction. This information would further indicate the catalytic site of the agarase. The ^13^C-NMR spectrum of the reaction products did not indicate a signal at approximately 90 ppm which was typically observed when α-(1,3) linkages are hydrolyzed to form agaro-oligosaccharides (Lahaye et al. 1989). The main resonance signals of anomeric carbons were in accordance with neoagaro-oligosaccharides, In Fig. 5, signals at approximately 96 and 92 ppm were assigned to the α- and β-anomeric carbons in the reducing end of the 3-*O*-linked β-d-galactopyranose residue, respectively. Multiple signals were observed for the α/β-anomeric carbons of the 3-*O*-linked β-d-galactopyranose indicating that the reaction products consisted of several neoagaro-oligosaccharides with different degrees of polymerization. These data suggest the hydrolytic products were neoagarotetraose and neoagarohexaose, and that the recombinant agarase is a β-agarase.

**Fig. 6:**
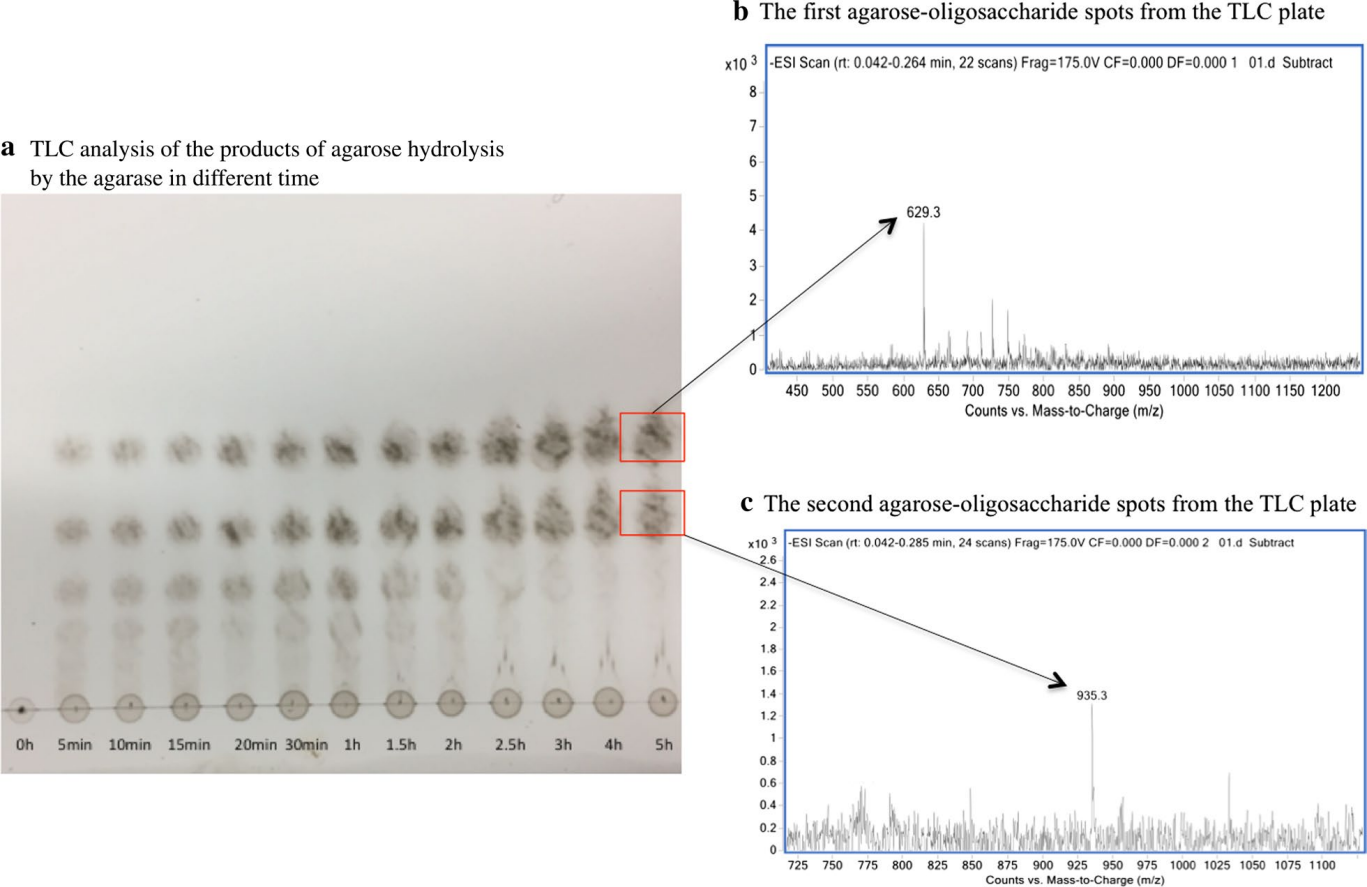
TLC analysis of the products of agarose hydrolysis by the agarase and determination of the molecular masses of the final products. **a** TLC analysis of the products of agarose hydrolysis by the agarase in different time. Hydrolysis reactions were conducted at 40 °C in 20 mM Tris–HCl buffer pH 7.2 containing 1% agarase substrate. Samples were taken at the indicated incubation times and analyzed by TLC as described in “Materials and methods section”. Determination of molecular masses of the two spots (**b** The first agarose-oligosaccharide spots from the TLC plate and **c** The second agarose-oligosaccharide spots from the TLC plate) recovered from the TLC plate showed that the final products of the reaction were neoagarotetraose and neoagarohexaose

To determine the hydrolytic pattern of the recombinant agarase, a time course of its activity was carried out by TLC. The agarase initially produced agarose-oligosaccharides of various lengths, which were progressively converted into smaller oligomers. Mass spectrometry analyses of the agarose-oligosaccharide spots extracted from the TLC plate indicated that the final reaction products are neoagarotetraose (Fig. 6b) and neoagarohexaose (Fig. 6c). These results confirm the recombinant agarase is an endo-type-β-agarase that produces neoagarotetraose and neoagarohexaose.

## Discussion

*Microbulbifer* sp. Q7 was isolated from the gut of sea cucumber. According to the results of genomic analysis, seven agarase-encoding sequences and five alginate lyase-encoding sequences were found (Yang et al. 2017). A few studies have shown that *Microbulbifer* plays a critical role in carbon recycling from marine biomass by degrading complex polysaccharides such as agar, carrageenan and alginate (Weiner et al. 2008). *Microbulbifer* has been isolated from rotten brown alga (Zhu et al. 2016), seawater (Sun et al. 2014) and mangrove forests (Mai et al. 2016; Moh et al. 2017). However, the seaweed-degrading strain of *Microbulbifer* isolated from the sea cucumber gut has not been previously reported. This study shows that the gut of sea cucumber is a source of seaweed polysaccharide degrading bacteria. Meanwhile, there were few reports on the agarase-encoding gene from *Microbulbifer* (Ohta et al. 2004). In our studies *ID2563* has the highest homology with other agarases in the NCBI database among the seven agarase-encoding genes founded from *Microbulbifer* sp. Q7 genome. Protein BLAST results showed that the agarase encoded by *ID2563* was novel, and had the highest sequence identity of 85% with the agarase from *M. agarilyticus* (BAE06228.1).

Soluble recombinant protein was successfully expressed in *E. coli*. Agarase found in both the intracellular and extracellular fractions were active. The total enzyme activity was 4.99 U/mL in fermentation medium, 65.73% of which was found in the agarase from the extracellular fraction under the action of the wild-type signal peptide. The signal peptide was composed of 19, mostly hydrophobic, amino acids (M_1_-K_2_-T_3_-T_4_-Q_5_-G_6_-A_7_-L_8_-A_9_-A_10_-L_11_-V_12_-F_13_-S_14_-T_15_-P_16_-L_17_-M_18_-A_19_). The previous study has showed the wild-type signal sequence can play an effective role on the secretion of recombinant protein in *E. coli* (Liu et al. 2014). However, the wild-type signal peptide is generally removed (Kim et al. 2010) or substituted with a secretion signal peptide (Kaewthai et al. 2010) before inserting the gene into the expression vector to facilitate overexpression. There is no general rule for selecting a proper signal sequence for recombinant proteins that will guarantee its secretion (Choi and Lee 2004). In this study, the wild-type signal peptide was retained and the recombinant agarase was successfully secreted by the *E. coli*. Thus, the wild-type signal peptide sequence from *Microbulbifer* may be useful for constructing secretory expression vectors.

Similar to most of the GH16 β-agarases (Aoki et al. 1990; Jung et al. 2017; Lin et al. 2017), the optimal temperature for extracellular agarase was around 40 °C. Its optimal pH was 6.0, which was slightly acidic in comparison with most of the previously reported β-agarases. The pH stability of agarase and its tolerance to high concentrations of ions and other chemical regents are important factors for its use in industrial applications. The agarase was stable between pH 6.0 and 9.0, which was a much wider range than most of the previously reported agarases, including those from *Alteromonas* sp. E-1 (Kirimura et al. 1999), *Vibrio* sp. AP-2 (Aoki et al. 1990), *Thalassospira profundimonas* fst-13007 (Zeng et al. 2016), *Gayadomonas joobiniege* G7 (Jung et al. 2017) and *Aquimarina agarilytica* ZC1 (Lin et al. 2017). These characteristics greatly improve the application potential of this agarase. The agarase was highly inhibited by Cu^2+^, Zn^2+^ and Fe^3+^. However, 5 mM Na^+^ and Fe^2+^ slightly improved its activity. Moreover, agarase activity was enhanced by 41% in the presence of β-mercaptoethanol, suggesting the catalytic site may contain thiols (Fu et al. 2008).

Sequence analysis indicated that the agarase belongs to the GH16 family. Thin layer chromatography, mass spectrometry and ^13^C-NMR analysis of the hydrolytic products further confirmed the conclusion. The final products of the recombinant agarase were neoagarotetraose and neoagarohexaose, a characteristic of the GH16 family agarases such as the agarase from *Zobellia galactanivorans* Dsij (Allouch et al. 2003), *Agarivorans* sp. LQ48 (Long et al. 2010) and *Flammeovirga* sp. SJP92 (Dong et al. 2016). The final products were concentrated in the supernatant of the maximum precipitation, which accounts for 90.47% of the total degradation products. Mass spectrometry analyses indicated the final products were primarily composed of neoagarotetraose and neoagarohexaose. Previous studies have suggested that the neoagarotetraose has many biological functions, such as anti-oxidative properties (Wu et al. 2010), whitening effects (Jang et al. 2009) and prebiotic properties (Hu et al. 2006). Thus, the recombinant agarase provides an efficient tool to produce the functional neoagar-oligosaccharides with potential applications in the cosmetic, food and pharmaceutical industries.

## Abbreviations

*E. coli*: *Escherichia coli*
DNS: 3,5-dinitrosalicylic acid
SDS-PAGE: sodium dodecyl sulfate polyacrylamide gel electrophoresis
ESI–MS: electrospray ionization mass spectrometry
^13^C-NMR: carbon-13 nuclear magnetic resonance
TLC: thin layer chromatography
